# Why do infants need out-of-hospital emergency medical services? A retrospective, population-based study

**DOI:** 10.1186/s13049-020-00816-8

**Published:** 2021-01-07

**Authors:** Jelena Oulasvirta, Heini Harve-Rytsälä, Mitja Lääperi, Markku Kuisma, Heli Salmi

**Affiliations:** 1 Division of Anesthesiology; Department of Anesthesiology, Intensive Care and Pain Medicine, University of Helsinki and Helsinki University Hospital, HUS, P.O. Box 340, FI-00029 Helsinki, Finland; 2 Department of Emergency Medicine and Services, University of Helsinki and Helsinki University Hospital, HUS, P.O.Box 340, FI-00029 Helsinki, Finland; 3 New Children’s Hospital, University of Helsinki and Helsinki University Hospital, HUS, P.O. Box 347, FI-00029 Helsinki, Finland

**Keywords:** Ambulance, Infant, Emergency department, Emergency medical services, Pediatric emergency care, Prehospital emergency care

## Abstract

**Background:**

The challenges encountered in emergency medical services (EMS) contacts with children are likely most pronounced in infants, but little is known about their out-of-hospital care. Our primary aim was to describe the characteristics of EMS contacts with infants. The secondary aims were to examine the symptom-based dispatch system for nonverbal infants, and to observe the association of unfavorable patient outcomes with patient and EMS mission characteristics.

**Methods:**

In a population-based 5-year retrospective cohort of all 1712 EMS responses for infants (age < 1 year) in Helsinki, Finland (population 643,000, < 1-year old population 6548), we studied 1) the characteristics of EMS missions with infants; 2) mortality within 12 months; 3) pediatric intensive care unit (PICU) admissions; 4) medical state of the infant upon presentation to the emergency department (ED); 5) any medication or respiratory support given at the ED; 6) hospitalization; and 7) surgical procedures during the same hospital visit.

**Results:**

1712 infants with a median age of 6.7 months were encountered, comprising 0.4% of all EMS missions. The most common complaints were dyspnea, low-energy falls, and choking. Two infants died on-scene. The EMS transported 683 (39.9%) infants. One (0.1%) infant died during the 12-month follow-up period. Ninety-one infants had abnormal clinical examination upon arrival at the ED. PICU admissions (*n* = 28) were associated with young age (*P* < 0.01), a history of prematurity or problems in the neonatal period (*P* = 0.01), and previous EMS contacts within 72 h (*P* = 0.04). The adult-derived dispatch codes did not associate with the final diagnoses of the infants.

**Conclusions:**

Infants form a small but distinct group in pediatric EMS care, with specific characteristics differing from the overall pediatric population. Many EMS contacts with infants were nonurgent or medically unjustified, possibly reflecting an unmet need for other family services. The use of adult-derived symptom codes for dispatching is not optimal for infants. Unfavorable patient outcomes were rare. Risk factors for such outcomes include quickly renewed contacts, young age and health problems in the neonatal period.

## Background

Children form a minority group of the emergency medical services (EMS) contacts. The reported proportion of EMS contacts in pediatric patients varies between 4% in Finland [1], 5% in Canada [2] and 7% in Denmark, Korea and United States [3–5]. However, children have unique needs in a healthcare setting and EMS has to address these needs [6]. The Academic Emergency Medicine Consensus Conference on “Aligning the Pediatric Emergency Medicine Research Agenda to Reduce Health Outcome Gaps” identified organization and administration of pediatric EMS as one of high-priority research issues [7].

EMS personnel have repeatedly reported experiencing challenges and feeling anxious when attending to pediatric patients [8–10]. Discomfort and anxiety seem to translate into true patient safety hazards, as significant medication errors [10, 11], non-systematic evaluation [12, 13], and other challenges [14, 15] in the emergency care of small children have been reported. Education, protocol development, mental aids, and encouragement for systematic evaluation have been proposed to address the challenges in pediatric emergency care [1, 14–16].

It has previously been shown that infants are overrepresented in pediatric EMS responses [1, 15, 17]. It has also been reported that the challenges that EMS personnel face when attending to children are most pronounced when attending to very young, nonverbal children [11, 13]. As infants differ markedly from older children, protocols based on studies conducted on general pediatric populations or even derived from adults, may not be suitable for infants. Because dispatch protocols are often symptom-based [3, 18] they may be difficult to apply to infants expressing themselves nonverbally. Thus, we sought to investigate EMS contacts with infants in detail.

The primary aim of this study was to describe the characteristics of the EMS contacts in the infant population. The secondary aims were to examine the use of the present symptom-based dispatch system for nonverbal infants, and to observe the occurrence of unfavorable patient outcomes and their associations with patient and EMS mission characteristics.

## Methods

### Study design and population

This was a population-based study that assessed retrospectively collected data from out-of-hospital and in-hospital patient records. We included all out-of-hospital ambulance responses for infants (age < 1 year) between 1 January 2013 and 31 December 2017 in Helsinki, Finland. Ambulance responses to out-of-hospital deliveries were excluded, as babies born out-of-hospital represent a very specific patient group not comparable to other EMS contacts with infants, and out-of-hospital births occur rarely in the study area [19].

### Setting

Helsinki is the capital and the largest city of Finland (population 643,000; < 1-year-old population 6548 in 2017). There were 6566 infants born in Helsinki in 2017 [20]. Finland is a Nordic welfare state with a publicly financed universal healthcare system, including free public prenatal clinics for pregnant women and ‘well-baby clinics’ for children aged 0 to 6 years. Prenatal and well-baby clinics offer parent training, and all families may contact their own community health nurse with problems and questions concerning child healthcare. Thus, advice and healthcare for infants are, in principle, easily available regardless of the socioeconomic status of the family.

Helsinki University Hospital (HUH) provides all pediatric out-of-hospital emergency care, pediatric secondary, and tertiary emergency department (ED) care and is responsible for the only pediatric intensive care unit (PICU) in the study area. Private-care providers and other public sector units offer some primary-level healthcare for children, but infants with altered medical state or requiring ambulance transport for medical reasons (or both) are referred to HUH pediatric EDs. Because of the centralized out-of-hospital and in-hospital pediatric emergency care, the data covers all ambulance responses in the study population.

All emergency calls from the study area are dialed to the same number (112). A professional emergency response center (ERC) operator first categorizes the leading complaint to form a symptom code and then determines a priority class from A to D following a formal national questionnaire protocol [18]. Ambulances are then dispatched with the combination of symptom code and priority class. The same set of symptom codes and priority classes are used for all patients regardless of age. The questionnaire protocol is the same for both adults and children. In the case of a pediatric patient, the ERC operator may ask some additional questions.

In Helsinki, all out-of-hospital emergencies are responded to by HUH EMS Helsinki consisting of 18 ambulances and a medical supervisor unit staffed by emergency medical technicians and paramedics. There is also one physician-staffed rapid response vehicle on call. Personnel of all ambulance units have the option of consulting with the physician by phone or requesting the physician-staffed unit to the scene for assistance.

Not all pediatric patients in Finland are transported to hospital by ambulance [1, 15]. After appropriate examination and possible treatment, the ambulance personnel may decide that the patient does not require ambulance transport. After the decision, the personnel inform the patient or the caregivers on how to monitor and treat the condition, and on whether or when to visit healthcare services by other means of transport. The non-transport decision and the information given are documented in the electronic patient record system.

### Variables

We obtained data on all out-of-hospital EMS responses concerning infants (age < 1 year) from an electronic patient record system (Merlot Medi®, CGI Suomi Oy). Data on ED visits and details of hospitalization were obtained from the HUH in-hospital patient record system (Uranus®, CGI Suomi Oy). Descriptive variables examined included age, sex, time of the contact, symptom code and priority class for dispatching and transport, physiological measurements conducted both in out-of-hospital and at the ED settings (respiratory rate, respiratory work, oxygen saturation, heart rate, systolic blood pressure, level of consciousness, blood glucose, temperature), insertion of an intravenous line in out-of-hospital setting, all treatments required at the ED including any type of medications or respiratory support (i.e. supplementary oxygen, nasal high flow or continuous positive airway pressure); all surgical procedures during the same hospital visit, diagnoses set at the ED, and diagnoses before the EMS contact. The diagnoses were obtained as International Classification of Diseases-10 (ICD-10) codes [21]. Demographics were obtained from Statistics Finland [20].

To evaluate the validity of using the same symptom codes for infants and adults during the dispatch process, we chose three symptom codes, “dyspnea”, “seizure” and “choking” which were common in the study population, and which could explicitly be related to the symptoms of specific ICD-10 diagnoses. We examined if these symptom codes were more related to those specific diagnoses than to other diagnoses. If the dispatch protocol was valid to recognize the presentation of symptoms in infants, the symptom codes should be associated with specific diagnoses. The full list of diagnoses related to the symptom codes is shown in the Additional file 1.

To explore the unfavorable patient outcomes, we chose to study 12-month mortality after the EMS contact. Based on our previous studies, mortality in this population was expected to be low [1, 15]. Therefore, we also studied the following outcomes reflecting the severity of the condition of the infants transported to the ED: 1) PICU admissions during the same hospital visit; 2) medical state of the infant upon arrival to the ED, which was judged based on the first documented physiologic measurements and the verbal evaluation by the physician; and categorized to “good” (all measurements and the presentation documented as normal) or “other than good” (any abnormal measurement or presentation documented); 3) any medication or respiratory support given at the ED (i.e. supplementary oxygen, nasal high flow or continuous positive airway pressure); 4) hospitalization during the same hospital visit; and 5) surgical procedures during the same hospital visit. Surgical procedures included endoscopy and bronchoscopy, but minor procedures performed at the ED (e.g. suturing a minor wound or inserting a nasogastric tube) were excluded.

For those infants not transported by ambulance after the EMS contact, we examined whether they visited the ED within 72 h of the initial non-transport decision. In case of such a visit, we examined the secondary outcomes 1 to 5 as mentioned above.

### Statistical analysis

Due to the lack of previous studies, we were unable to make estimates on the incidence of outcomes for power analysis. Thus, we chose a follow-up period of 5 years as a clinically relevant period in which no major changes (e.g. new significant protocols or changes in the hospital processes) were implemented. Continuous variables are presented as the median and the interquartile range (IQR) and categorical variables as frequencies and percentages (%). Multivariate analyses were not included due to the small number of observations in most of the outcomes, which would most likely lead to overfitting in regression models especially when adjusting with potential cofounders. Hence, a simpler approach with Mann-Whitney U test for continuous variables (age) and Fisher’s test for categorical comparisons (all outcomes as well as descriptive variables other than age) were utilized. Risk plots were constructed using the locally estimated scatterplot smoothing (LOESS) method with ggplot2 package [22]. The seasonal distribution and time-of-day variation were analyzed visually. Two-tailed *P*-values were used with P-values below 0.05 considered significant. The analyses were performed using R version 3.6.2 (R Core Team (2018) Vienna, Austria [23]).

## Results

During the study period there were 401,372 ambulance responses leading to a patient contact in Helsinki. Of these, 1712 (0.4%) concerned infants (children < 1 year). Thus, the incidence of EMS contacts with infants was 0.11/1000 inhabitants/year in the study area. Two infants (0.1%) died on-scene or were dead upon arrival of the EMS. Nineteen infants had two or more EMS contacts within 72 h. Of the 1710 infants encountered alive, 1027 (60.1%) were not transported by ambulance and 183 (17.8%) were advised by ambulance personnel to visit the ED by other means of transport. Of the 1027 infants not transported by ambulance, 194 (19.0%) visited the ED within 72 h of the initial EMS contact. Of these ED visits, 106 (54.6%) were advised by ambulance personnel. Infants were seldom dispatched in the highest priority class (A), but a higher dispatch priority (A or B) was more common in the infants who were eventually transported to the ED, as compared to those requiring no transport.

**Fig. 1:**
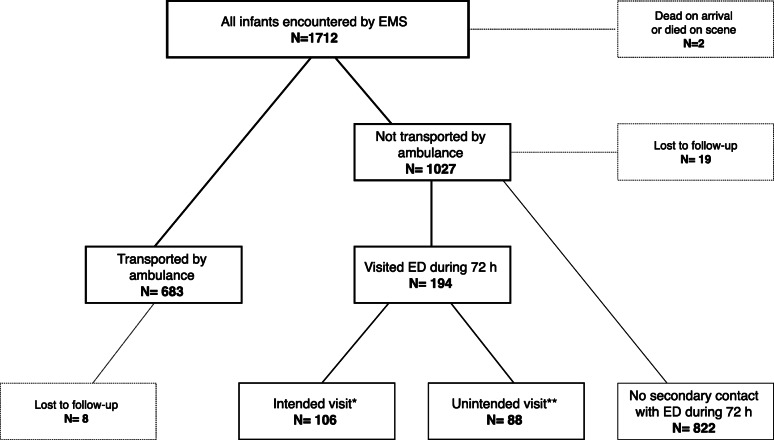
Patient flowchart. ED = Emergency department. EMS = Emergency medical services. * Patient was not transported by ambulance but advised by ambulance personnel to visit ED by means of transport other than ambulance. ** Patient was not transported by ambulance but visited ED within 72 h self-imposed

Of the 1710 infants encountered alive, 27 (1.6%) could not be followed in detail because of incomplete personal details. Most of these were tourists without a Finnish identification number. Eight were initially transported by ambulance and thus the in-hospital data concerning the visit directly following the ambulance transport were available. The patient flow is described in Fig. 1.

**Fig. 2:**
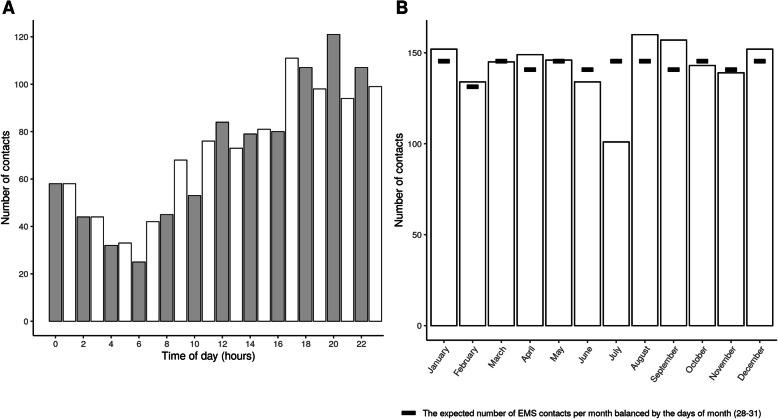
Distribution of the emergency medical contacts by hour and month. A = EMS contact per hour. B = EMS contacts per month. EMS = Emergency medical services

The median age of the infants was 6.7 months (IQR 2.81–9.47). A total of 896 (52.3%) were boys and 402 (23.5%) had a native language other than one of the two national languages, Finnish or Swedish. There were more contacts in late evening, with a peak at 20:00. Seasonal variation was not observed. Contacts according to the time of day and time of year are illustrated in Fig. 2.

The out-of-hospital measurements are presented in Table 1. No out-of-hospital measurements were conducted for 256 (15.0%) of the infants.

**Table 1:** Characteristics of the study population and EMS contacts. The infants transported and not transported by ambulance were compared using Mann-Whitney U test for age and Fisher’s test for other variables

	All infants encountered alive *N = 1710*	Infants not transported by ambulance *N = 1027*	Infants transported by ambulance *N = 683*	*P*-value
Age (months) median (IQR)	6.7 (2.81–9.47)	7 (3.4–9.53)	6 (2.07–9.29)	**< 0.01**
Sex (males)	894 (52.4%)	536 (52.3%)	358 (52.5%)	0.96
SpO_2_ measured OOH	957 (56.0%)	531 (51.7%)	426 (62.4%)	**< 0.01**
Heart rate measured OOH	1007 (58.9%)	573 (55.8%)	434 (63.5%)	**< 0.01**
Respiratory rate measured OOH	577 (33.7%)	352 (34.3%)	225 (32.9%)	0.60
Temperature measured OOH	994 (58.1%)	533 (51.9%)	461 (67.5%)	**< 0.01**
Blood glucose measured OOH	343 (20.1%)	149 (14.5%)	194 (28.4%)	**< 0.01**
Blood pressure measured OOH	445 (26.0%)	235 (22.9%)	210 (30.7%)	**< 0.01**
GCS measured OOH	498 (29.1%)	327 (31.8%)	171 (25.0%)	**< 0.01**
No measurements OOH	256 (15.0%)	171 (16.7%)	85 (12.4%)	**0.02**
I.v. access inserted OOH	2 (0.1%)	0 (0.0%)	2 (0.3%)	0.16
Lungs auscultated OOH	561 (32.8%)	344 (33.5%)	217 (31.8%)	0.49
Time of day				0.46
06:00–14:00	465 (27.2%)	281 (27.4%)	184 (26.9%)	
14:00–22:00	770 (45.0%)	451 (43.9%)	319 (46.7%)	
22:00–06:00	475 (27.8%)	295 (28.7%)	180 (26.4%)	
Most common symptom codes				**< 0.01**
“Dyspnea”	471 (27.5%)	262 (25.5%)	209 (30.6%)	
“Fall (low energy)”	322 (18.8%)	241 (23.5%)	81 (11.9%)	
“Choking”	144 (8.4%)	117 (11.4%)	27 (4.0%)	
Priority class for dispatch				**< 0.01**
A	55 (3.2%)	16 (1.6%)	39 (5.7%)	
B	789 (46.1%)	416 (40.5%)	373 (54.6%)	
C	820 (48.0%)	558 (54.3%)	262 (38.4%)	
D	46 (2.7%)	37 (3.6%)	9 (1.3%)	
Most common diagnoses at ED^b^				0.56
J06.9 “Common cold”	83 (24.6%)	21 (26.9%)	62 (23.9%)	
J04.0 “Laryngitis”	54 (16.0%)	17 (21.8%)	37 (14.3%)	
R56.8 “Seizure”	61 (18.1%)	12 (15.4%)	49 (18.9%)	
R68.1 “Infant with nonspecific symptoms”	49 (14.5%)	8 (10.3%)	41 (15.8%)	
Z03.9 “Observation”	49 (14.5%)	11 (14.1.%)	38 (14.7%)	
S06.0 “Concussion”	41 (12.2%)	9 (11.5%)	32 (12.4%)	
1-year mortality	1 (0.1%)	0 (0.0%)	1 (0.1%)	0.40
PICU admissions	28 (3.2%)	2 (1.0%)^a^	26 (3.8%)	0.06
Medical state other than good upon arrival at ED	91 (11.1%)^a^	11 (5.7%)^a^	80 (12.8%)	**0.01**
Any medication or respiratory support given at ED	386 (47.2%)^a^	82 (42.3%)^a^	304 (48.8%)	0.12
Hospitalization	336 (19.8%)^a^	51 (26.3%)^a^	285 (41.7%)	**< 0.01**
Surgical procedures during the same visit	18 (2.1%)^a^	2 (1.0%)^a^	16 (2.3%)	0.39

*SpO*_*2*_ Peripheral oxygen saturation, *ED* Emergency department, *GCS* Glasgow Coma Scale, *I.v.* Intravenous, *OOH* Out-of-hospital, *PICU* Pediatric intensive care unit

^a^In proportion to those infants who visited ED within 72 h

^b^ The full names of the ICD-10 diagnoses are shown in the Additional file 1

One (0.1%) of the infants encountered alive died during the 12-month follow-up period. The death was due to a chronic illness and did not have a causal connection to the EMS contact. Of all 1710 infants encountered alive, 877 were seen in the ED. Of these, 683 (77.9%) were directly transported by ambulance, 726 (83%) were mentioned to be in a good medical state at arrival and 386 (44%) were medicated or given respiratory support (e.g. oxygen or inhalations) at the ED. In total, 60 infants were transported by ambulance to primary healthcare clinics and the reason for ambulance transport was merely logistical or social, not medical. For these 60 infants, the medical state upon arrival and possible medication were not available for study purposes; there were no hospitalizations, PICU admissions, or need for surgical procedures in this patient group. In the entire study population, there were 28 PICU admissions, 336 hospitalizations, and 18 surgical procedures following an ED visit that occurred within 72 h of the initial EMS contact. All descriptive variables and outcomes are presented and compared between the transported and non-transported infants in Table 1.

The association of specific ICD-10 diagnoses and the three evaluated symptom codes for dispatching “Dyspnea”, “Seizure” and “Choking” are presented in Table 2.

**Table 2:** Associations between ICD-10 diagnoses and symptom codes for dispatching

Symptom code for dispatch	Related diagnoses^a^	Nonspecific diagnoses^b^	Other diagnoses^c^	No ED contact
“Dyspnea” *N = 472*	180 (38.1%)	55 (11.7%)	47 (10.0%)	190 (40.3%)
“Seizure” *N* = 117	68 (58.1%)	8 (6.8%)	23 (19.7%)	18 (15.4%)
“Choking” *N* = 144	10 (6.9%)	10 (6.9%)	4 (2.8%)	120 (83.3%)

% referring to the proportion of each code

*ED* Emergency department, *ICD* International Classification of Diseases

^a^ICD-10 diagnoses related to the symptom. The full list of related diagnoses is shown in the Additional file 1

^b^ ICD-10 diagnoses not related to any medical symptom or diagnosis, including R68.1, Z00, Z01, Z02, and Z03. See Additional file 1

^c^All diagnoses other than ^a^ and ^b^

**Fig. 3:**
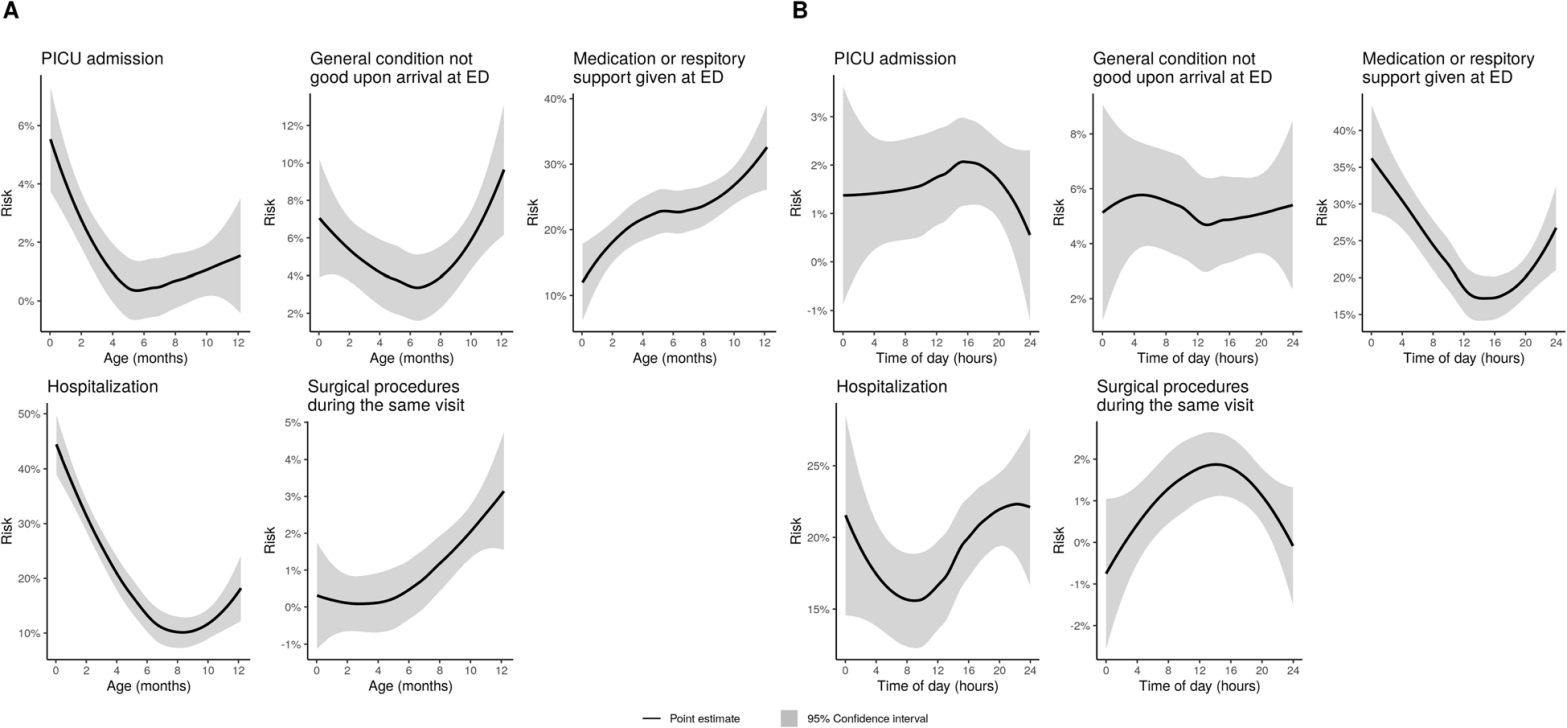
Associations between secondary outcomes, age, and time of emergency medical services contact. A = Associations between secondary outcomes and age. B = Associations between secondary outcomes and time of the EMS contact. ED = Emergency Department. EMS = Emergency medical services

As there was only one death during the 12-month follow-up, we were not able to use this outcome for comparison. Young age was associated with a greater risk for PICU admissions (1.79 months (IQR 0.74–8.88) among those with PICU admission vs. 6.73 months (IQR 2.97–9.48) among those without PICU admission, *P* < 0.01), and hospitalizations (3.49 months (IQR 1.30–8.17) vs. 7.10 months (IQR 3.97–9.63), *P* < 0.001). Older infants were more prone to require medication or respiratory support at ED (7.57 months (IQR 4.25–10.22) vs. 6.43 months (IQR 2.53–9.30), *P* < 0.01) and surgical procedures (9.75 months (IQR 8.75–10.82) vs. 6.63 months (IQR 2.8–9.43), *P* < 0.01). A previous ICD-10 diagnosis from chapter P representing conditions related to prematurity or problems during the neonatal period was associated with PICU admissions, medical state other than good upon arrival, and hospitalizations. The associations between the studied variables and the secondary outcomes are presented in Tables 3, 4, and 5 and in Fig. 3.

**Table 3:** Proportions (%) of patients with the most frequent symptom codes for dispatching among the patients **with** and **without** each secondary outcome. I.e. 39.3% of patients requiring PICU admission and 27.3% of patients not requiring PICU admission had “Dyspnea” as symptom code. Comparisons between the proportions **with** and **without** were made using Fisher’s test

	“Urgent dispatch before symptom specific code known”	“Dyspnea”	“Fall low energy”	“Choking”	“Seizure”	“Slow deterioration of medical state”	“Allergic reaction”
*% of all (N = 28) patients* ***with*** *PICU admissions*	21.4%	39.3%	0.0%	6%	7.1%	0.0%	0.0%
% of all (*N* = 1682) patients **without** PICU admission	5.0%	27.3%	19.0%	8.6%	6.8%	6.9%	4.2%
	***P*** **< 0.01**	***P*** **= 0.02**	***P*** **= 0.01**	*P* = 0.51	*P* = 0.72	*P* = 0.25	*P* = 0.63
% of all (*N = 91)* patients with medical state **other than good** upon arrival at ED	8%	42.9%	4.4%	1.1%	15.4%	2.2%	4.4%
% of all (*N = 1619)* patients with medical state **good** upon arrival at ED or ED care not required	5.1%	26.7%	19.6%	8.8%	6.4%	7.1%	4.1%
	*P* = 0.14	***P*** **< 0.01**	***P*** **< 0.01**	***P*** **< 0.01**	***P*** **< 0.01**	*P* = 0.08	*P* = 0.79
% of all (*N = 386)* patients **with** any medication or respiratory support given at ED	7.0%	38.3%	6.2%	1.3%	13.2%	7.0%	7.5%
% of all (*N = 1324)* patients **without** any medication or respiratory support given at ED	4.8%	24.3%	22.4%	10.6%	5.0%	6.8%	3.2%
	*P* = 0.09	***P*** **< 0.01**	***P*** **< 0.01**	***P*** **< 0.01**	***P*** **< 0.01**	*P* = 0.91	***P*** **< 0.01**
% of all (*N = 336)* patients **with** hospitalization^a^	11.6%	36.3%	6.8%	3.3%	14.0%	3.6%	2.4%
% of all (*N = 1363)* patients **without** hospitalization^a^	3.7%	25.3%	21.6%	9.8%	5.1%	7.6%	4.6%
	***P*** **< 0.01**	***P*** **< 0.01**	***P*** **< 0.01**	***P*** **< 0.01**	***P*** **< 0.01**	***P*** **= 0.01**	*P* = 0.07
% of all (*N = 18)* patients **with** surgical procedures^b^ during the same visit^a^	5.6%	5.6%	0.0%	38.9%	0.0%	0.0%	0.0%
% of all (*N = 1692)* patients **without** surgical procedures^b^ during the same visit^a^	5.3%	27.7%	18.9%	8.1%	6.9%	6.9%	4.2%
	*P* = 1.00	***P*** **= 0.03**	***P*** **= 0.03**	***P*** **< 0.01**	*P* = 0.63	*P* = 0.63	*P* = 1.00

*ED* Emergency department, *PICU* Pediatric intensive care unit, *EMS* Emergency medical services

^a^ During the same hospital visit beginning within 72 h after the initial EMS contact

^b^ Excluding minor procedures performed at the ED, e.g. insertion of a nasogastric tube

**Table 4:** Proportions (%) of patients with each variable among the patients **with** and **without** each secondary outcome. I.e. 7.1% of patients requiring PICU admission and 1.0% of patients not requiring PICU admission had a previous EMS contact. Comparisons between the proportions **with** and **without** were made using Fisher’s test

	Previous EMS contact within 72 h	No measurements conducted by EMS	Native language other than Finnish or Swedish	Previous ICD10 P-diagnosis^a^	Previous ICD10 Q-diagnosis^b^
*% of all (N = 28) patients* ***with*** *PICU admissions*	*7.1%*	*3.6%*	*25.0%*	*35.7%*	*14.3%*
% of all (*N* = 1682) patients **without** PICU admission	1.0%	15.0%	29.2%	15.7%	6.3%
	***P*** **= 0.04**	*P* = 0.11	*P* = 0.83	***P*** **= 0.01**	*P* = 0.10
% of all (*N = 91)* patients with medical state **other than good** upon arrival at ED	*0.0%*	*4.4%*	*20.9%*	*25.3%*	*19.8%*
% of all (*N = 1619)* patients with medical state **good** upon arrival at ED or ED care not required	1.2%	15.7%	29.5%	5.4%	5.7%
	*P* = 0.62	***P*** **< 0.01**	*P* = 0.10	***P*** **= 0.02**	***P*** **< 0.01**
% of all (*N = 386)* patientsc **with** any medication or respiratory support given at ED	*2.1%*	*8.5%*	*29.4%*	*17.4%*	*11.1%*
% of all (*N = 1324)* patients **without** any medication or respiratory support given at ED	0.8%	16.6%	29.1%	15.6%	16.1%
	*P* = 0.05	***P*** **< 0.01**	*P* = 0.90	*P* = 0.43	*P* = 0.75
% of all (*N = 336)* patients **with** hospitalization^c^	*2.1%*	*9.5%*	*26.3%*	*22.9%*	*11.0%*
% of all (*N = 1363)* patients **without** hospitalization^c^	0.9%	16.1%	29.9%	14.3%	5.4%
	*P* = 0.08	***P*** **< 0.01**	*P* = 0.20	***P*** **< 0.01**	***P*** **< 0.01**
% of all (*N = 18)* patients **with** surgical procedures^d^ during the same visit^d^	*0.0%*	*22.2%*	*11.1%*	*11.1%*	*11.1%*
% of all (*N = 1692)* patients **without** surgical procedures^d^ during the same visit^d^	1.1%	14.7	29.4%	16.1%	6.4%
	***P*** **< 0.05**	*P* = 0.32	*P* = 0.12	*P* = 0.75	*P* = 0.33

*ED* Emergency department, *EMS* Emergency medical services, *ICD* International Classification of Diseases, *PICU* Pediatric intensive care unit

^a^Certain conditions originating in the perinatal period

^b^Congenital malformations, deformations, and chromosomal abnormalities

^c^During the same hospital visit beginning within 72 h after the initial EMS contact

^d^Excluding minor procedures performed at the ED, e.g. insertion of a nasogastric tube

**Table 5:** Proportions (%) of patients encountered at different times of day. Fisher’s test was used to compare the proportions of patients **with** and **without** each secondary outcome inside the time categories

	Time of the day		
	6:00–14:00	14:00–22:00	22:00–6:00
*% of all (N = 28) patients* ***with*** *PICU admissions*	21.4%	60.7%	17.9%
% of all (*N* = 1682) patients **without** PICU admission	27.3%	44.6%	28.1%
	*P* = 0.26		
% of all (*N = 91)* patients with medical state **other than good** upon arrival at ED	25.3%	44.0%	30.8%
% of all (*N = 1619)* patients with medical state **good** upon arrival at ED or ED care not required	27.3%	45.1%	27.6%
	*P* = 0.78		
% of all (*N = 386)* patients **with** any medication or respiratory support given at ED	24.6%	38.6%	36.8%
% of all (*N = 1324)* patients **without** any medication or respiratory support given at ED	28.0%	46.7%	25.3%
	***P*** **< 0.01**		
% of all (*N = 336)* patients **with** hospitalization^a^	21.7%	50.3%	28.0%
% of all (*N = 1363)* patients **without** hospitalization^a^	28.6%	43.5%	27.9%
	***P*** **= 0.02**		
% of all (*N = 18)* patients **with** surgical procedures^b^ during the same visit^a^	44.4%	55.6%	0.0%
% of all (*N = 1692)* patients **without** surgical procedures^b^ during the same visit^a^	27.1%	44.7%	28.2%
	***P*** **= 0.01**		

*ED* Emergency department, *PICU* Pediatric intensive care unit

^a^During the same hospital visit beginning within 72 h after initial EMS contact

^b^Excluding minor procedures performed at the ED, e.g. insertion of a nasogastric tube

## Discussion

In this population-based study, we observed that infants encountered by the EMS had specific EMS mission characteristics differing from EMS contacts in the general population, and from those in the general pediatric population. The dispatch priority classes set by the ERC associated with the urgency of the condition, but the dispatch symptom codes did not relate to the final diagnoses at the ED. The 12-month mortality was low, as well as the the other studied unfavorable patient outcomes. Infants had a high non-transportation rate in the EMS, and often received a nonspecific diagnosis at the ED. Only few infants required PICU admission, any medications or respiratory support, hospitalization or surgical procedures during the same hospital visit, or were not in good medical state upon arrival at the ED.

The complaints leading to EMS contacts were different in infants, as compared to those reported in the overall pediatric population [15, 24]. Whereas dyspnea is among the leading causes for EMS contacts in children overall, infants had additional frequent EMS contacts due to choking. These contacts often resulted in the child not being transported to the hospital, as the causative foreign object of the airway had already been removed. Traffic accidents and poisonings, common causes of EMS contacts in older children [24, 25], were rare in infants.

The very high proportion of non-transport (60%) of infants in our study is striking. The figure is considerably higher than previously reported in children overall and even higher than in our previous studies on the general pediatric population in the same area [1, 15]. As EMS personnel have reported anxiety when attending to small children [8, 9, 14], it is logical to assume that for the sake of safety, the EMS personnel would be more inclined to transport the younger the child is. The diurnal distribution of the EMS contacts and the absence of seasonal variation also suggest that many of the contacts for infants did not have a specific medical cause (Fig. 2). The EMS contacts for infants were clustered to afternoon and evening, with 45% occurring between 14:00 to 20:00 and peaking around 20:00. This is not explained by any medical reason. Even if diurnal variation in the incidence of some common conditions (e.g. febrile convulsions [26]) played a role, this finding could arise from the fact that other services (e.g. community health nurse consultation) are only available during limited office hours. If the peak in EMS contacts in the evening was due to medical causes, this should have been reflected in the condition and treatment of the infants at ED. However, being contacted in the later hours was not associated with unfavorable patient outcomes (Table 5). In addition, if most complaints had been due to medical causes, the usual pediatric seasonal variation [27, 28] in the incidence of acute illnesses should have been observed. It is possible that instead of medical problems, many EMS contacts with infants represent complex social challenges. The low rate of unfavorable patient outcomes, including 12-month mortality rate as low as 1/1710 among infants requiring EMS care seems to support this assumption. Thus, we conclude that the EMS responses for infants were often medically unjustified.

It can be argued that challenges in emergency care for infants support the current practice of keeping a low threshold for activating the EMS for infants. Nevertheless, our results support the possibility of optimizing the use of EMS by addressing other services for children. When no medical intervention or even no medical visit is needed, EMS is not the most efficient, adequate, or capable healthcare provider to respond to the need for counseling and advice in families with small children. Instead, implementation of other low-threshold health and counseling services, open after office hours, should be studied and encouraged [29–32].

As EMS personnel have reported anxiety and uncertainity when attending to small children [8, 9, 14], our findings may be relieving. At the ED in a tertiary-level pediatric unit, a significant number of infants were discharged with nonspecific ICD-10 codes, such as R68.1 “infant with nonspecific symptoms” or Z03.9 “observation”. This highlights the challenges even specialized medical personnel encounter when attending to young, nonverbal children with a short medical history. Thus, it is vital to encourage EMS providers to comprehensively examine the infant regardless of the possibly transient symptoms described by the caregivers. Indeed, even though EMS personnel did study vital signs more often than in the previously reported studies [13, 24, 33]; only heart rate, temperature and peripheral oxygen saturation reached over 50% coverage.

The efficacy of dispatch protocols in guiding appropriate use of EMS for pediatric patients has not been confirmed [34]. To our knowledge, none of the current dispatch protocols has been validated in infants. We noticed that the current dispatch priorities A-D were consistent with the urgency of the condition, as the proportion of ambulance transport to hospital increased with each class of dispatch priority (Table 1), and with the symptom codes “dyspnea”, “urgent dispatch before symptom specific code known”, and “seizure”, the infants were more likely to experience unfavorable secondary outcome (Table 3). By comparing the symptom codes and the final diagnoses at the ED, we detected that the symptom codes of the current dispatch protocol did not seem to represent the symptoms of nonverbal infants. The use of unsuitable symptom codes for dispatching may be misleading in infants, because the dispatch code may steer the approach of the EMS personnel before attending the scene. Therefore, we suggest that the functionality of adult-driven symptom codes for dispatching in infants should be critically evaluated. The dispatch process for infants should target at noticing the symptoms that are recognizable for the ERC operator and typical for out-of-hospital emergencies in infants.

We also identified easily recognizable patient characteristics associated with an increased risk for unfavorable outcomes. Infants with repeated EMS contacts over a short time period (72 h) had an increased risk for PICU admissions. In daily practice, repeated calls may be interpreted as representing a low parental threshold for calling an ambulance. Instead, ERC operators and EMS personnel should regard quickly renewed contacts as a true indicator for more serious illness in infants. We found that diagnoses related to prematurity and problems in the neonatal period were associated with a higher risk for hospitalization and PICU admission. This is consistent with previous studies showing that children with problems in the neonatal period also have higher health service utilization in later infancy [35, 36]. Age had a different association with different outcomes (Fig. 3). Although older infants had a greater risk for surgical procedures, it is noteworthy that young infants were more often hospitalized and admitted to PICU.

The strength of our study is that we had full coverage of the EMS encounters in the population and a relatively large cohort of infants with respect to the thorough study of outcome variables in every child. In addition, few infants were lost to follow-up. Our study also has several limitations. As mortality and PICU admission rates in pediatric populations in high-income countries are low, we had to choose less robust secondary outcomes to evaluate the quality of EMS care for infants. Due to the lack of previous studies, we were unable to make estimates on the incidence of outcomes for power analysis, and multivariate analyses were not included due to small number of observations in most of the outcomes. Thus, we had to settle for a convenience follow-up period of 5 years. Our results represent associations and not causalities. In addition, our results from one city may not be directly generalizable to other areas. Finally, our data search did not cover emergency calls without EMS response, and thus complete sensitivity of the dispatch protocol could not be established. Also, we did not have access to the patient records of the primary healthcare or private providers. Ultimately, all infants that require hospitalization or surgical procedures are referred to the clinics of the HUH.

## Conclusions

Infants are a minority in EMS care, forming 0.4% of EMS contacts. The characteristics of EMS contacts with infants are different from those in adults, or from the general pediatric population. The use of current adult-derived symptom codes for dispatching does not seem to be optimal for categorizing the symtoms of infants. Unfavorable patient outcomes, including 12-month mortality, were rare in infants encountered by the EMS. Risk factors for such outcomes include quickly renewed contacts, young age and health problems in the neonatal period. Many EMS contacts with infants did not seem urgent or were even medically unjustified, possibly reflecting an unmet need for counseling and supportive services for families.

## Supplementary Information

**Additional file 1.** Full names of the International Classification of Diseases (ICD-10) codes referred in the study.

## Data Availability

The datasets generated and analyzed during the current study are not publicly available as they are based on patient registers. These datasets are available from the corresponding author on reasonable request in the form in which all individual data is de-identified and any data cannot be combined such that an individual case can be recognized. Each request will require permission from the Helsinki University Hospital.

## Abbreviations

ED: Emergency department
EMS: Emergency medical services
ERC: Emergency response center
HUH: Helsinki University Hospital
ICD: International Classification of Diseases
PICU: Pediatric intensive care unit
