# Supplementary material for: Why do infants need out-of-hospital emergency medical services? A retrospective, population-based study
Source: Scand J Trauma Resusc Emerg Med. 2021 Jan 7;29:13. doi: 10.1186/s13049-020-00816-8 (PMC7789394; doi:10.1186/s13049-020-00816-8)
Supplement: Supplementary file 1 — Additional file 1. Full names of the International Classification of Diseases (ICD-10) codes referred in the study. [file 13049_2020_816_MOESM1_ESM.docx]

Additional file 1: Full names of the International Classification of Diseases (ICD-10) codes referred to in the study

*ICD-10 codes for pediatric conditions that could have presented with* ***dyspnea.*** *The association of symptom code “dyspnea” with being diagnosed with these codes at the emergency department has been studied.*

| J14 |  |  | Pneumonia due to *Haemophilus influenzae* |  |  |  |  |  |  |  |
| --- | --- | --- | --- | --- | --- | --- | --- | --- | --- | --- |
| J15 |  |  | Bacterial pneumonia, not elsewhere classified |  |  |  |  |  |  |  |
| J16 |  |  | Pneumonia due to other infectious organisms, not elsewhere classified |  |  |  |  |  |  |  |
| J17 |  |  | Pneumonia in diseases classified elsewhere |  |  |  |  |  |  |  |
| J18 |  |  | Pneumonia, organism unspecified |  |  |  |  |  |  |  |
| J20 |  |  | Acute bronchitis |  |  |  |  |  |  |  |
| J21 |  |  | Acute bronchiolitis |  |  |  |  |  |  |  |
| J36 |  |  | Peritonsillar abscess |  |  |  |  |  |  |  |
| J37 |  |  | Chronic laryngitis and laryngotracheitis |  |  |  |  |  |  |  |
| J38.4 |  |  | Oedema of larynx |  |  |  |  |  |  |  |
| J38.5 |  |  | Laryngeal spasm |  |  |  |  |  |  |  |
| J39.0 |  |  | Retropharyngeal and parapharyngeal abscess |  |  |  |  |  |  |  |
| J39.1 |  |  | Other abscess of pharynx |  |  |  |  |  |  |  |
| J40 |  |  | Bronchitis, not specified as acute or chronic |  |  |  |  |  |  |  |
| J41 |  |  | Simple and mucopurulent chronic bronchitis |  |  |  |  |  |  |  |
| J42 |  |  | Unspecified chronic bronchitis |  |  |  |  |  |  |  |
| J44 |  |  | Other chronic obstructive pulmonary disease |  |  |  |  |  |  |  |
| J45 |  |  | Asthma |  |  |  |  |  |  |  |
| J46 |  |  | Status asthmaticus |  |  |  |  |  |  |  |
| J86 |  |  | Pyothorax |  |  |  |  |  |  |  |
| J90 |  |  | Pleural effusion, not elsewhere classified |  |  |  |  |  |  |  |
| P25 |  |  | Interstitial emphysema and related conditions originating in the perinatal period |  |  |  |  |  |  |  |
| P26 |  |  | Pulmonary haemorrhage originating in the perinatal period |  |  |  |  |  |  |  |
| P27 |  |  | Chronic respiratory disease originating in the perinatal period |  |  |  |  |  |  |  |
| P28 |  |  | Other respiratory conditions originating in the perinatal period |  |  |  |  |  |  |  |
| R06 |  |  | Dyspnoea |  |  |  |  |  |  |  |
| R09 |  |  | Other symptoms and signs involving the circulatory and respiratory systems |  |  |  |  |  |  |  |

*ICD-10 codes for pediatric conditions that could have presented with* ***seizures.*** *The association of symptom code “seizure” with being diagnosed with these codes at the emergency department has been studied.*

| G01 |  |  | Meningitis in bacterial diseases classified elsewhere |  |  |  |  |  |  |  |
| --- | --- | --- | --- | --- | --- | --- | --- | --- | --- | --- |
| G02 |  |  | Meningitis in other infectious and parasitic diseases classified elsewhere |  |  |  |  |  |  |  |
| G03 |  |  | Meningitis due to other and unspecified causes |  |  |  |  |  |  |  |
| G05 |  |  | Encephalitis, myelitis and encephalomyelitis in diseases classified elsewhere |  |  |  |  |  |  |  |
| G06 |  |  | Intracranial and intraspinal abscess and granuloma |  |  |  |  |  |  |  |
| G40 |  |  | Epilepsy |  |  |  |  |  |  |  |
| G41 |  |  | Status epilepticus |  |  |  |  |  |  |  |
| G45 |  |  | Transient cerebral ischaemic attacks and related syndromes |  |  |  |  |  |  |  |
| R25.2 |  |  | Cramp and spasm |  |  |  |  |  |  |  |
| R25.8 |  |  | Other and unspecified abnormal involuntary movements |  |  |  |  |  |  |  |
| R56 |  |  | Convulsions, not elsewhere classified |  |  |  |  |  |  |  |
| P52 |  |  | Intracranial nontraumatic haemorrhage of fetus and newborn |  |  |  |  |  |  |  |
| P90 |  |  | Convulsions of newborn |  |  |  |  |  |  |  |
| P91 |  |  | Other disturbances of cerebral status of newborn |  |  |  |  |  |  |  |
| I60 |  |  | Subarachnoid haemorrhage |  |  |  |  |  |  |  |
| I61 |  |  | Intracerebral haemorrhage |  |  |  |  |  |  |  |
| I62 |  |  | Other nontraumatic intracranial haemorrhage |  |  |  |  |  |  |  |
| I63 |  |  | Cerebral infarction |  |  |  |  |  |  |  |
| I65 |  |  | Occlusion and stenosis of precerebral arteries, not resulting in cerebral infarction |  |  |  |  |  |  |  |
| I67 |  |  | Other cerebrovascular diseases |  |  |  |  |  |  |  |
| I68 |  |  | Cerebrovascular disorders in diseases classified elsewhere |  |  |  |  |  |  |  |
| A86 |  |  | Unspecified viral encephalitis |  |  |  |  |  |  |  |
| A87 |  |  | Viral meningitis |  |  |  |  |  |  |  |
| A88 |  |  | Other viral infections of central nervous system, not elsewhere classified |  |  |  |  |  |  |  |
| A89 |  |  | Unspecified viral infection of central nervous system |  |  |  |  |  |  |  |

*ICD-10 codes which could be used if the infant was diagnosed as having or having had a* ***foreign body in the airway.*** *The association of symptom code “choking” with being diagnosed with these codes at the emergency department has been studied.*

| T17 | Foreign body in respiratory tract |  |  |  |
| --- | --- | --- | --- | --- |
| T18.0 | Foreign body in mouth |  |  |  |
| T18.1 | Foreign body in oesophagus |  |  |  |
| T18.2 | Foreign body in stomach |  |  |  |
| J69.0 | Pneumonitis due to food and vomit |  |  |  |
| J69.8 | Pneumonitis due to other solids and liquids |  |  |  |
| J70.8 | Respiratory conditions due to other specified external agents |  |  |  |
| J70.9 | Respiratory conditions due to unspecified external agent |  |  |  |
| R06.1 | Stridor |  |  |  |
| R06.2 | Wheezing |  |  |  |

*Unspecific ICD-10 codes. These codes were seen as representing challenges in establishing a diagnosis in an infant at the emergency department.*

| R68.1 |  | Nonspecific symptoms peculiar to infancy |  |  |  |  |  |  |  |  |  |
| --- | --- | --- | --- | --- | --- | --- | --- | --- | --- | --- | --- |
| R68.8 |  | Other specified general symptoms and signs |  |  |  |  |  |  |  |  |  |
| P92 |  | Feeding problems of newborn |  |  |  |  |  |  |  |  |  |
| R63 |  | Symptoms and signs concerning food and fluid intake |  |  |  |  |  |  |  |  |  |
|  |  |  |  |  |  |  |  |  |  |  |  |
| Z00 |  | General medical examination |  |  |  |  |  |  |  |  |  |
| Z01 |  | Other special examinations and investigations of persons without complaint or reported diagnosis |  |  |  |  |  |  |  |  |  |
| Z02 |  | Examination and encounter for administrative purposes |  |  |  |  |  |  |  |  |  |
| Z03 |  | Medical observation and evaluation for suspected diseases and conditions |  |  |  |  |  |  |  |  |  |

*The most common ICD-10 codes given at the emergency department.*

| J06.9 | Acute upper respiratory infection, unspecified |  |  |  |  |
| --- | --- | --- | --- | --- | --- |
| J04.0 | Acute laryngitis |  |  |  |  |
| R56.8 | Other and unspecified convulsions |  |  |  |  |
| R68.1 | Nonspecific symptoms peculiar to infancy |  |  |  |  |
| Z03.9 | Observation for suspected disease or condition, unspecified |  |  |  |  |
| S06.0 | Concussion |  |  |  |  |
